# Human vitreous concentrations of citicoline following topical application of citicoline 2% ophthalmic solution

**DOI:** 10.1371/journal.pone.0224982

**Published:** 2019-11-14

**Authors:** Carmela Carnevale, Gianluca Manni, Gloria Roberti, Alessandra Micera, Luca Bruno, Andrea Cacciamani, Romeo Altafini, Luciano Quaranta, Luca Agnifili, Lucia Tanga, Ivano Riva, Francesco Oddone

**Affiliations:** 1 IRCCS-Fondazione Bietti, Rome, Italy; 2 DSCMT, University of Rome Tor Vergata, Rome, Italy; 3 Ophthalmology Clinic, Dolo Hospital, Venezia, Italy; 4 Department of Surgical & Clinical, Diagnostic and Pediatric Sciences, Section of Ophthalmology, University of Pavia-IRCCS Fondazione Policlinico San Matteo, Pavia, Italy; 5 Ophthalmology Clinic, Department of Medicine and Aging Science, University G. d'Annunzio of Chieti-Pescara, Chieti, Italy; University of Florida, UNITED STATES

## Abstract

**Purpose:**

To evaluate the presence and concentration of citicoline and its metabolites (choline, cytidine and uridine) in the vitreous body in human eyes after topical application of an ophthalmic solution of citicoline 2%, in vivo.

**Methods:**

Twenty-one subjects affected by epiretinal membrane with surgical indication for pars-plana vitrectomy underwent treatment with 1 drop 3 times/day of a solution of citicoline 2%, 0.2% high molecular weight hyaluronic acid and 0.01% benzalkonium chloride (OMK1, Omikron Italia s.r.l., Rome, Italy) 14 days before surgery and 2 hours prior to surgery. Five additional patients served as controls and received an OMK1 vehicle solution without citicoline. The vitreous samples were taken at the beginning of the pars-plana vitrectomy and analyzed for qualitative/quantitative determination of vitreous concentration of citicoline and its metabolites by means of high performance liquid chromatography.

**Results:**

The overall mean concentration of citicoline in patients treated with citicoline 2% solution was 406.72 ± 52.99 μg/mL, while the mean concentration of choline, cytidine and uridine was 180.88 ± 41.49 μg/mL, 44.45 ± 10.19 μg/mL and 330.41 ± 75.8 μg/mL, respectively. The concentration of citicoline in phakic eyes (n = 13, 366.61 ± 129.61 μg/mL) was lower compared to that found in pseudophakic eyes (n = 8, 435.89 ± 131.42 μg/mL) and the difference was not statistically significant. The concentration of citicoline in the control eyes was 45.66 ± 26.36 μg/mL, while the concentration of choline, cytidine and uridine were 17.21 ± 9.93 μg/mL, 6.24 ± 3.6 μg/mL and 172.80 ± 99.76 μg/mL, respectively.

**Conclusion:**

Citicoline can reach the human vitreous in high concentration when administered in ophthalmic solution. This evidence contributes to the build-up of the pyramid of the evidences required for determining the role of citicoline administered in ophthalmic formulation in retinal and optic nerve neurodegenerative diseases.

## Introduction

Citicoline (cytidine-5’-diphosphocholine or CDP-Choline) is a mononucleotide composed by ribose, pyrophosphate, cytosine and choline. It is considered an intermediate in the biosynthesis of phosphatidylcholine (PDC), one of the most important phospholipids of the cell membranes, and the precursor of the neurotransmitter acetylcholine in the central nervous system [1]. In addition, citicoline increases tyrosine hydroxylase activity and inhibits dopamine reuptake thus increasing brain neurotransmitters like dopamine, noradrenaline and serotonin [2].

Citicoline is quickly metabolized to choline and cytidine. Plasmatic cytidine is then deaminated by cytidine deaminase to uridine. Uridine is the precursor of uridine triphosphate in the brain, cytidine triphosphate at neuronal level and citicoline [3].

It is well known that intramuscular and oral citicoline has a protective role in cerebrovascular and neurodegenerative diseases like stroke [4], Alzheimer’s and Parkinson’s disease [5,6].

Furthermore, several studies focused on the effect of oral citicoline on retinal ganglion cells (RGCs) function in ocular diseases like amblyopia [7,8], optic neuropathies [9,10] and glaucoma [11, 12].

Citicoline is also currently available in ophthalmic solution for topical treatment in eye-drops. It has been recently documented in an animal experimental model using high performance liquid chromatography (HPLC), that citicoline in ophthalmic formulation reaches the vitreous (using as vehicles high molecular weight hyaluronic acid (HA) and benzalkonium chloride (BAK)) [13]. This evidence plays a key role in the understanding of the potential action of this molecule, when administered topically, directly on the retinal targets that are morpho-functionally involved in the glaucomatous disease that are RGCs and their axons.

An improvement in RGCs function as assessed by electrophysiology has been also documented in the clinical phase of the same study in a small series of glaucomatous patients after two months of treatment with citicoline eye-drops [13].

Nevertheless, despite these interesting findings, to the best of our knowledge, there is no evidence in the literature showing that citicoline in topical formulation can reach the human vitreous body from where it is expected to interact with the retinal targets involved in the glaucomatous neurodegeneration.

The primary aim of the present study was to fill this gap in the currently available knowledge by evaluating the presence and concentration of citicoline and its metabolites (choline, cytidine and uridine) in the vitreous body in vivo in human eyes after topical eye-drops administration.

Secondary aims were the comparison of citicoline and its metabolites concentration in the vitreous of phakic and pseudophakic eyes and the correlations with age and ocular biometric parameters including central corneal thickness (CCT) and corneal endothelial cells density (ECD).

## Material and methods

This cross-sectional study was conducted at the Glaucoma Research Unit of the IRCCS Fondazione Bietti (Rome, Italy). The study was approved by the IFO-Bietti Ethical Committee (Registry N.61/17/FB) and conducted according to the principles of the Declaration of Helsinki as well as the international rules for biosamples’ management. This study was registered on ClinicalTrials.gov (NCT04003090). Informed written consent was obtained from all participants, including both study conductance and permission to use and store human biological material for research purposes.

Patients affected by epiretinal membrane (ERM) with surgical indication of 23 gauge minimally invasive pars-plana vitrectomy (PPV) associated or not with cataract surgery were included in the study. To be enrolled patients had to be older than 18 years and able to understand and sign the written informed consent.

Patients were excluded from the study in case of any laser treatments and ocular surgery in the past 6 months, hypersensitivity to the active ingredients used in the study, other systemic or ocular diseases different from ERM that could affect the outcome of the study, aphakia or previous complicated cataract surgery, intraocular lens (IOL) in the anterior chamber, treatment with systemic citicoline or other potential neuroprotective agents in the past 6 months, pregnancy or breastfeeding.

All patients underwent a comprehensive ophthalmologic evaluation including measurement of best-corrected visual acuity obtained using ETDRS charts, slit-lamp biomicroscopy, intraocular pressure measurement with Goldmann applanation tonometry and fundus dilated indirect ophthalmoscopy.

Additionally, all patients had central retinal thickness measurements with spectral-domain optical coherence tomography (Spectralis SD-OCT; Heidelberg Engineering, Heidelberg, Germany) associated with CCT (Sirius Scheimpflug camera, C.S.O., Italy) and ECD (Nidek Specular Microscope CM-530, Nidek technology, Padova, Italy) evaluations.

Patients included in the study were instructed to start the treatment with an ophthalmic solution of citicoline 2% eye-drops, 0.2% HA and 0.01% BAK (OMK1, Omikron Italia s.r.l., Rome, Italy, chemical properties: pH 6.5–7.5, specific gravity/relative density: 1.0112, viscosity: around 5–10 cPs, osmolality: 270–330 mOsm/kg) with the following administration scheme: 1 drop for 3 times/day over a total time of 14 days before surgery and 1 drop 2 hours prior to the surgery.

Furthermore, 5 patients matching the same inclusion and exclusion criteria received an OMK1 vehicle solution (without citicoline) administered with the same scheme and herein used as control. Vitreous concentrations of citicoline and its metabolites were investigated also in these patients.

### Surgical technique

All patients underwent a 23G standard 3-port PPV, (R-Evolution CR; Optikon 2000, Inc., Rome, Italy), with posterior vitreous detachment induction, ERM removal, internal limiting membrane (ILM) staining with 0,25 g/l of brilliant blue-G with (Brilliant Peel ^™^, Geuder, Germany) and creation of 360° ILM flap around the central macular area. All phakic patients underwent combined phacoemulsification with IOL implantation in-the-bag. No cases suffered intraoperative complications or capsular ruptures.

### Vitreous sampling, total protein analysis and pre-treatments

The vitreous sample was taken at the beginning of the PPV for all patients by the same surgeon (A.C.) according to a standard procedure.

At surgery, the vitreous sample was collected with the infusion line closed into a sterile 5 mL syringe connected directly to the vitreous cutter to avoid contamination with blood or other substances. After the aspiration was completed, the syringe was removed from the vitrectomy probe and the surgery was conducted following the clinical indication.

Samples were coded and quickly delivered to the Research’s laboratory Unit placed in the same building, as well as analysed for protein quality/quantity, aliquoted and stored at -70°C until evaluation. Vitreous samples were subjected to volume quantification and spectrophotometrical analysis of total proteins by using an ultra-fast UV/vis spectrometer equipped with LVis Plate (spectroSTAR nano; BMG LABTECK GmbH, Allmendgrün, Ortenberg) that provides qualitative and quantitative analysis of total proteins. Appropriate protein aliquots (20 μL) were prepared to allow all determinations, from an initial total protein pretreatment (Thermo Fisher, Milan, Italy).

### HPLC analysis: Mobile/Stationary phases, standard curves and analysis

Chromatographic separation was performed at ambient temperature by using the C18 column (Ascentis Express C18, 15 cm × 3 mm, 5 μm particle size; Phenomenex, Milan, Italy). The mobile phase included 0.1 M potassium dihydrogen phosphate and acetonitrile (ACN) in a 30:70 (v/v), all as HPLC grade. Other chemicals and MilliQ water were of analytical grade and guaranteed for HPLC analysis. Samples (10 μL injected by using Hamilton HPLC syringe; 22s gauge—25 μL volume) were isocratically eluted at 0.5 mL /min flow rate. All analysis was carried out by using the Shimadzu HPLC instrument equipped with UV detector at 274 nm (LC-2010HT version 3.10; Shimadzu LC solution, Kyoto, Japan). Preliminary analysis was carried out to set up all the method in terms of linearity, accuracy, repeatability and precision. The running time was set at 10 min after a preliminary analysis over 25 min. This procedure allowed the development and validation of a HPLC method in reverse phase which involved column analysis (stationary phase) and the evaluation of a suitable mobile phase to be used in the study.

Standard solutions were of high pure quality and prepared according to a standard procedure for HPLC analysis: citicoline sodium salt (> 98% purity, 33818-15-4, Kyowa Hakko Bio Co., Ltd.); choline chloride (>99% purity; C7017, Sigma Aldrich), cytidine (>99% purity; C122106, Sigma Aldrich) and uridine (Bioultra >99% purity; U6381, Sigma Aldrich). Stocks were produced (scalar dilutions: 50, 30, 15, 5 and 0 μg/mL) in ultrapure HPLC water (Sigma, Milan, Italy) and freshly produced for each set of experiments. Positive and negative controls were produced and verified at each daily analysis, with respect to the specific column. Auto check and calibrations were carried out at the beginning of each analysis. All detections were carried out in duplicate and repeated three times to validate reproducibility. Caffein (C6035; 10ng/mL in ddw; Sigma-Aldrich) was used as internal control. Standard curves were obtained by using ratio of peak area (calculated at retention time) and standard concentrations. Areas were calculated by software at each retention time by using the amplitude and height parameters. Regression curve was defined and intercept, slope as well as correlation coefficient (polynomial grade 3) were produced and used for calculating the analytic concentration of each target with respect to eventual concentration in each sample. Every retention time characterised the peak of elution, as defined at the beginning of the calibration activity. Detection limits were defined at the lower concentration yielding a signal-to-noise. To reduce inter and intra-day as well as other potential bias, at least two evaluations were performed and repeated at least three different times, and two operators provided acquisitions (L.B. and A.M.). A Coefficient of variation <10% was used to validate results.

### Statistical analysis

Continuous variables were expressed as mean ± standard deviation if normally distributed or as median and interquartile range if non-normally distributed. Categorical variables were expressed as frequencies and percentages. The independent t-test or Mann-Whitney U test were used to compare continuous variables according to their distribution. Relationships between the biochemical concentration of citicoline in vitreous samples and age, CCT and ECD were explored by linear regression analysis. A *p* value <0.05 was considered statistically significant. Statistical analysis was performed using JMP Ver. 9.0.1 (SAS Institute Inc.).

## Results

Twenty-one eyes of 21 subjects were enrolled in the study and received citicoline 2% ophthalmic solution. Demographic and clinical characteristics of these patients are summarized in Table 1.

**Table 1:** Demographic and clinical characteristics of patients treated with citicoline 2% ophthalmic solution.

	N = 21 Mean ± SD
Sex (M/F)	11/10
Age (years)	71.6 ± 7.3
BCVA (logMar)	1.0 ± 0
IOP (mmHg)	19 ± 5
CCT (μm)	549 ± 24
ECD (cells/mm^2^)	2480 ± 292

SD = standard deviation; M = male; F = female; BCVA = best corrected visual acuity; IOP = intraocular pressure; CCT = central corneal thickness; ECD = corneal endothelial cell density

Eight eyes (38%) were pseudophakic and underwent the 23-gauge PPV whereas 13 eyes (62%) were phakic and performed a combined procedure of 23-gauge PPV and cataract phacoemulsification.

All patients self-administered citicoline 2% eye-drops until the day of surgery and no complications were encountered during the vitreous sampling procedures.

The chromatographic characteristics of vitreous were acquired at 274 nm and used to define the mobile phase. Both methanol and ACN33 were tested at beginning and finally the best results were obtained with the ratio above reported (materials and methods section). The isocratic mode was achieved by using protein precipitation and/or standard preparation in a control vitreous, according to a standard procedure.

**Fig 1:**
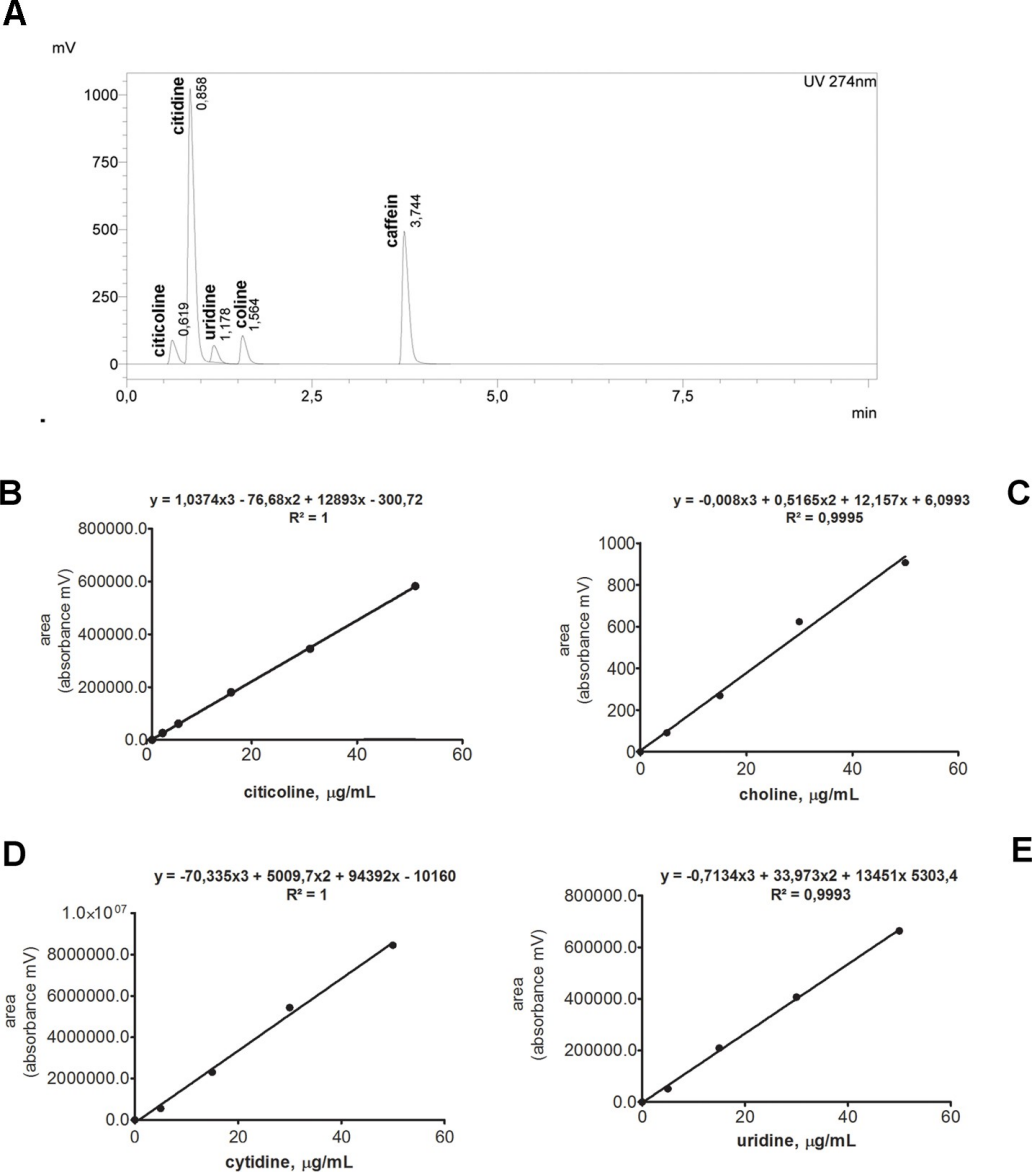
**(A-E). Representative chromatogram of a mixture of citicoline and metabolites.** Representative chromatogram of a mixture of citicoline and metabolites. (A) HPLC chromatogram obtained for standard mixture containing citicoline, choline, cytidine and uridine at concentration of 50 ng/mL. The retention times are reported close to each peak, as indicative of specificity of analysis. Peak purity data (areas) were used to produce a standard curve as shown in panels (B) for citicoline, (C) for choline, (D) for cytidine and (E) for uridine. The linearity curve for each analytics was confirmed by the R^2^ value of the polynomial curve (3^rd^ grade), as shown in each panel. Chromatogram parameters were as described in the materials and methods paragraph. In preliminary tests, a run time of 25 min was performed to assure the absence of other contaminants and to assess the background noise, and thereafter reduced to 10 min.

The concentrations of citicoline, choline, cytidine and uridine analytics were calculated by using the calibration curves produced in parallel. Fig 1 shows a representative HPLC chromatogram with peaks corresponding to the presence of citicoline and related metabolites in a control vitreous, produced for graphical representation.

The mean values of citicoline, choline, cytidine and uridine in the 5 controls treated with the vehicle were: 45.66 ± 26.36 μg/mL, 17.21 ± 9.93 μg/mL, 6.24 ± 3.6 μg/mL and 172.80 ± 99.76 μg/mL, respectively.

**Fig 2:**
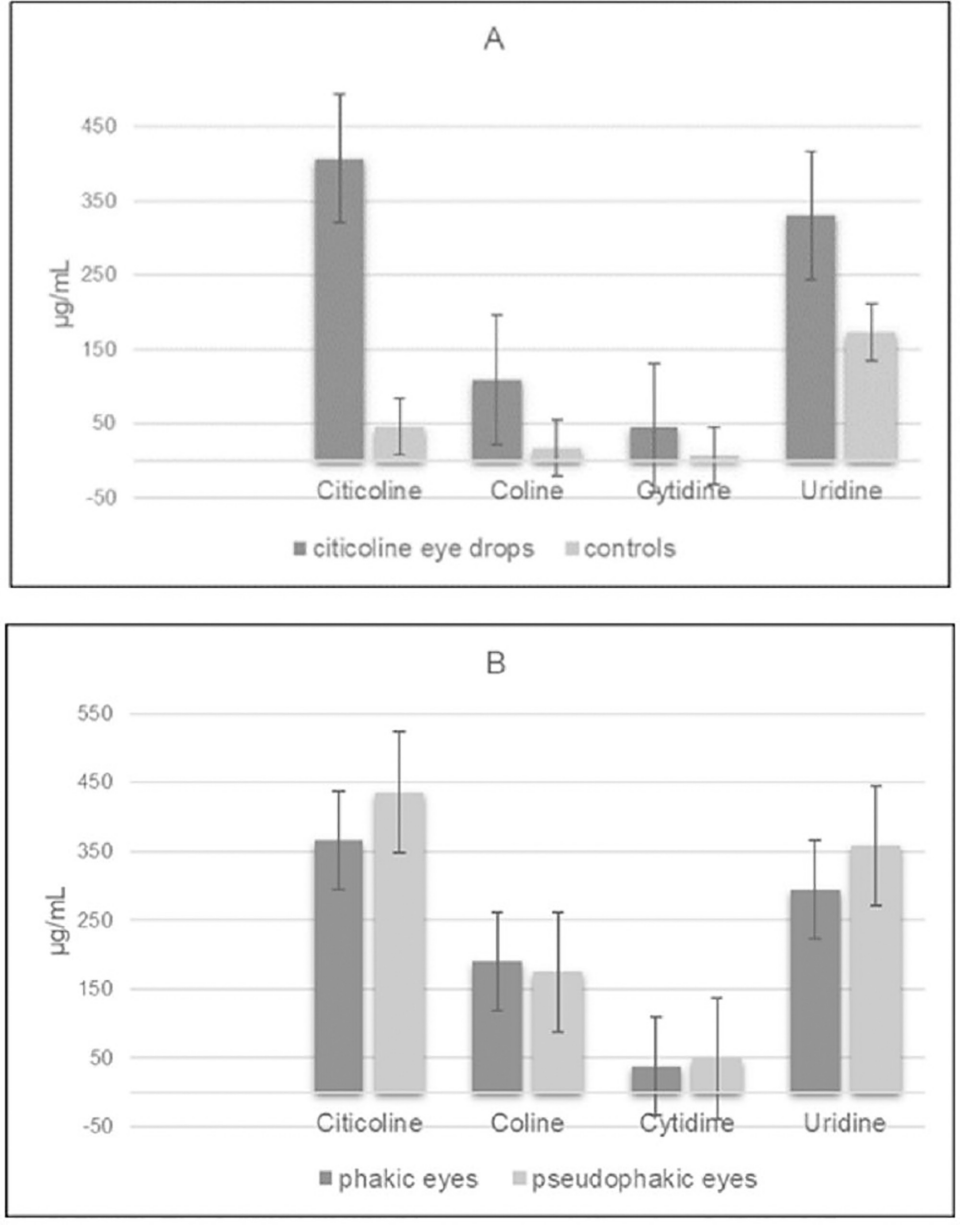
**(A-B). Vitreous concentration of citicoline and metabolites.** Citicoline and metabolites presence in human vitreous from control eyes (n = 5) and citicoline 2% treated eyes (n = 21). Samples were subjected to specific HPLC evaluations, after a protein extraction. (A) Citicoline, choline, cytidine and uridine values (μg/mL) in vitreous from control and citicoline 2% treated eyes. (B) Comparison between phakic (n = 13) and pseudophakic (n = 8) eyes in citicoline 2% treated eyes. Mann-Whitney U test T was used for statistical analysis.

An overall increased expression of citicoline and metabolites was quantified in vitreous samples collected from citicoline topically treated eyes (Fig 2A).

The overall mean concentration of citicoline was 406.72 ± 52.99 μg/mL, while the mean concentration of choline, cytidine and uridine was 180.88 ± 41.49 μg/mL, 44.45 ± 10.19 μg/mL and 330.41 ± 75.8 μg/mL, respectively.

Citicoline amount in vitreous of phakic eyes treated with citicoline 2% eye-drops was somewhat lower than that found in pseudophakic eyes, although the difference was not statistically significant (366.61 ± 129.61 μg/mL, n = 13, vs 435.89 ± 131.42 μg/mL, n = 8, p = 0.31; Fig 2B). In addition, no significant differences of metabolite concentrations were found between phakic and pseudophakic vitreous samples (choline: 189.31±66.93 μg/mL *vs*. 174.74±52.68 μg/mL, p = 0.44; cytidine: 37.43±13.23 μg/mL *vs*. 49.36±14.88 μg/mL, p = 0.42; uridine: 294.74±104.20 μg/mL *vs*. 358.15±107.98 μg/mL, p = 0.45) (Fig 2B).

**Fig 3:**
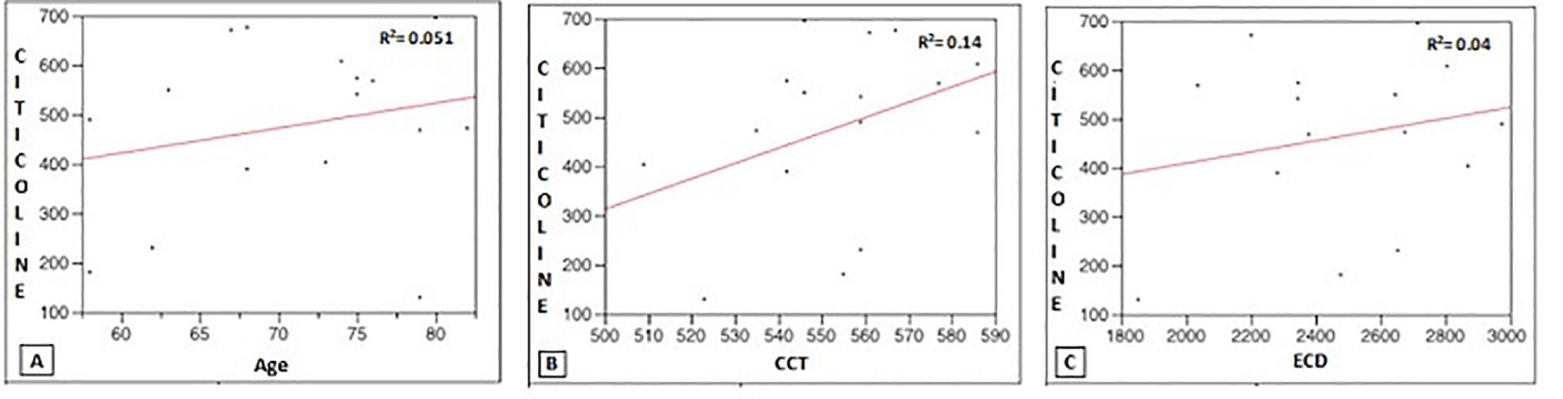
Correlation between vitreous citicoline values and baseline age, CCT and ECD. Citicoline amounts (μg/mL) were correlated to baseline (A) age (years), (B) CCT (μm) and (C) ECD (cells/mm2). Linear regression is shown for each analysis.

Finally, there was no significant relationship between citicoline concentration into the vitreous samples and the following patient parameters: age (R^2^ = 0.051, *p* = 0.4), central corneal thickness (R^2^ = 0.14, *p* = 0.15) and corneal endothelial cell density (R^2^ = 0.04, *p* = 0.78) (Fig 3).

## Discussion

The main aim of the present study was to explore whether citicoline can reach the vitreous body in vivo in human eyes when administered at 2% concentration in eye-drop formulation. For this purpose, we enrolled a cohort of patients affected by ERM scheduled for PPV that were treated with 1 drop for 3 times a day of citicoline 2% eye-drop formulation for 14 days before surgery and 1 drop 2 hours prior to the surgery. The presence and concentration of citicoline and its metabolites choline, cytidine and uridine in the vitreous samples collected at the time of surgery were then analyzed by means of HPLC.

The main result of the present study is the finding of citicoline in high concentration in the vitreous body of human eyes after topical eye-drop administration, as well as an increased concentration of its metabolites. To the best of our knowledge, this is the first evidence in literature showing that citicoline delivered in eye-drop formulation can reach the vitreous body in human eyes. These results confirm the findings of a previously published work were citicoline was found after eye-drop topical administration in the vitreous body in animal models [13].

Citicoline sodium salt has a high molecular weight, equal to 510,31 Dalton. Usually molecules with this size may have difficulty to pass across the corneal epithelium and to reach the vitreous body. For these reasons the OMK1 solution was added with HA (0.2%) and BAK (0.01%). Benzalkonium chloride has been shown to acts as a corneal penetration enhancer as it relaxes the intercellular junctions of the corneal epithelium in a reversible manner [14]. HA, thanks to its viscoelastic properties, rises the viscosity of solution, increasing the contact time of the citicoline sodium salt solution with the ocular surface. The presence of both BAK and HA in OMK1 is likely to contribute to the penetration of the molecule of citicoline in the vitreous body.

The finding of the presence of citicoline in the vitreous body after topical administration is of clinical relevance since it represents a necessary, although not sufficient, condition to support a potential interaction of this molecule with retinal targets involved in glaucoma or in other retinal neurodegenerative diseases.

Citicoline is an endogenous compound that acts as an intermediate in the synthesis of membrane phospholipids and specifically of PDC which is a key component of cell membranes thus involved in cell homeostasis conservation [15]. Citicoline also acts as a precursor of the neurotransmitter acetylcholine in the nervous system [1] and when systemically administered it increases tyrosine hydroxylase activity and inhibits dopamine reuptake, thus increasing the levels of neurotransmitters like dopamine, noradrenaline and serotonin [2].

Based on this evidence, citicoline has been widely studied for its neuroprotective and neuro-enhancing role in neurological diseases such as stroke [4], brain injury [16], Alzheimer’s and Parkinson’s disease [5,17]. There is an increasing body of evidence that citicoline may play a role also in ophthalmological diseases such as amblyopia [7,8], optic neuropathies [9,10], glaucoma [18] and diabetic retinopathy [19]. Experimental *in vitro* and *in vivo* studies have shown that citicoline has a neuroprotective effect on damaged RGCs since it is able to neutralize excitotoxicity due to glutamate action [19,20,21]. In addition, citicoline contributes to enhance visual function by improving the catecholaminergic neurotransmission [22]. Most of the clinical knowledge about the functional action on the visual pathway of citicoline in glaucoma derives from the systemic use of this molecule administered either intramuscularly or in oral solution [23,24].

An eye-drop formulation of citicoline has been recently made available. This formulation is stable when stored at room temperature under 25°C. Biocompatibility studies were performed on the eye drops OMK1 during the development phase in order to evaluate, according to ISO 10993–1, cytotoxicity, allergic sensitization and eye irritation of the formulation confirming that the eye drop OMK1 is non-cytotoxic, non-sensitizing and non-irritating (MD OMK1, Technical File, Omikron Italia srl). Moreover the MD OMK1 has been registered in EU and Eurasia since 2011, without any side effects (Manufacturer Post-Marketing Surveillance).

In addition, the effects of this formulation on visual function have been explored in clinical settings. In a case-control prospective study, Parisi et al. explored the retinal function and the neural conduction along the visual pathway after treatment with citicoline eye-drops in 56 patients with open angle glaucoma [25]. The authors found an enhancement of the retinal bioelectrical responses (increase of pattern electroretinogram amplitude) with a consequent improvement of the bioelectrical activity of the visual cortex (shortening and increase of visual evoked potentials implicit time and amplitude, respectively) in patients treated with topical citicoline, suggesting that citicoline in this formulation can reach the vitreous and interact with the retinal targets [25].

An additional finding of our study was a statistically similar concentration of citicoline detected in the vitreous body of both phakic and pseudophakic eyes. Nevertheless, despite the vitreous concentration of citicoline was high in both groups, the somewhat numerically lower concentration found in phakic eyes (366.61 ± 129.61 μg/mL) compared to pseudophakic eyes (435.89 ± 131.42 μg/mL), although not reaching the statistical significance (p = 0.31), might be indicating that the integrity of the cristalline lens might play a subtle role in the bioavailability of this molecule in the vitreous body.

These results agree with a previous study by Takamura et al. where the authors analyzed the vitreous and aqueous humor concentrations of brimonidine topical ophthalmic solution 0.1% by means of liquid chromatography and tandem mass spectrometry in 24 patients undergoing PPV for idiopathic ERM or macular hole [26]. The authors reported no significant differences of brimonidine concentration in the vitreous between phakic and pseudophakic eyes [26].

Moreover, similarly to our results, Kent et al. [27], reported a slightly higher concentration of brimonidine in the vitreous of pseudophakic eyes compared to that found in phakic eyes, although the difference between the two groups was not statistically significant.

In the present study, the concentration of the citicoline metabolites choline, cytidine and uridine was found to be similar in phakic compared to pseudophakic eyes. Nevertheless, among the citicoline metabolites, we found a higher concentration of uridine compared to choline and cytidine. This finding could be explained by considering the well-known wide availability of uridine in human tissues, being a product of several biochemical pathways such as uracil metabolism [27].

In addition, we explored whether CCT could influence the penetration of citicoline into the eye and consequently its concentration in the vitreous body. The lack of relationship found between the concentration of citicoline and CCT (R^2^ = 0.14, *p* = 0.15) indicates that the thickness of the central cornea is not playing a significant role in the penetration of this molecule inside the eye. These results agree with previous works in which the relationship between CCT and the intraocular penetration of drugs in topical eye-drop formulation was explored [28,29].

In a study by Martinez-de-la-Casa et al. three groups of 20 patients each scheduled for cataract surgery were selected according to CCT (between 510 μm and 575 μm) and received one drop of 0.004% travoprost two hours before surgery [29]. A sample of humour aqueous was obtained performing a paracentesis with a 23G needle and was analysed. No significant differences emerged when concentrations of 0.004% travoprost were compared among groups.

Similar results were reported in a study by Spierer et al. that investigated the penetration of multiple administrations of vancomycin eye drops before cataract surgery in 58 patients with CCT between 458 μm and 635 μm [30]. Again, no significant difference of vancomycin concentration in the anterior chamber was found among patients enrolled in the study.

Furthermore, in our study neither ECD or age were found to be associated with the concentration of citicoline found in vitreous body suggesting that these factors do not influence the penetration of citicoline into the eye.

Among the strengths of the present study it should be highlighted that it involved a relatively large cohort of homogeneous patients and that vitreous sampling was performed by only one experienced surgeon thus limiting the variability of the results related to the sampling procedure. Among the weaknesses, we should mention the potential coelution issue related with the HPLC technique. Because of the speed of HPLC and its reliance on different polarities of compounds, two compounds with similar structure and polarities can exit the chromatography apparatus nearly the same time making the exact determination of which portion of the mixture eluted at what point difficult.

Neverthless, the 10-fold lower concentration of citicoline found in the vitreous body of patients treated only with the vehicle makes the higher concentration of citicoline found in treated patients unlikely related to a coelution issue that, if present, should have affected both groups in a similar manner.

In conclusion, in this study we report for the first time the presence of citicoline and its concentration in the vitreous body of human eyes in-vivo after topical administration of an ophthalmic solution. This evidence contributes to the build-up of the pyramid of the evidences required for determining the role of citicoline administered in ophthalmic formulation in retinal and optic nerve neurodegenerative diseases.
